# Application of ultrasensitive digital ELISA for p24 enables improved evaluation of HIV-1 reservoir diversity and growth kinetics in viral outgrowth assays

**DOI:** 10.1038/s41598-023-37223-9

**Published:** 2023-07-06

**Authors:** Yury V. Kuzmichev, Carol Lackman-Smith, Sonia Bakkour, Ann Wiegand, Michael J. Bale, Andrew Musick, Wendy Bernstein, Naomi Aronson, Julie Ake, Sodsai Tovanabutra, Mars Stone, Roger G. Ptak, Mary F. Kearney, Michael P. Busch, Elizabeth R. Wonderlich, Deanna A. Kulpa

**Affiliations:** 1 Division of Microbiology and Immunology, Emory National Primate Research Center, Emory University, Atlanta, GA USA; 2 Department of Infectious Disease Research, Southern Research, Frederick, MD USA; 3 Vitalant Research Institute, San Francisco, CA USA; 4 Department of Laboratory Medicine, University of California San Francisco, San Francisco, CA USA; 5 HIV Dynamics and Replication Program, NCI at Frederick, NIH, Frederick, MD USA; 6 Uniformed Services University, Bethesda, MD USA; 7 Walter Reed National Military Medical Center, Bethesda, MD USA; 8 U.S. Military HIV Research Program, Walter Reed Army Institute of Research, Silver Spring, MD USA; 9 Department of Pathology and Laboratory Medicine, Emory University School of Medicine, Atlanta, GA USA; 10 Present Address: Laboratory of Epigenetics and Immunity, Department of Pathology and Laboratory Medicine, Weill Cornell Medicine, New York, NY USA

**Keywords:** HIV infections, Retrovirus, Viral reservoirs, ELISA

## Abstract

The advent of combined antiretroviral therapy (cART) has been instrumental in controlling HIV-1 replication and transmission and decreasing associated morbidity and mortality. However, cART alone is not able to cure HIV-1 due to the presence of long-lived, latently infected immune cells, which re-seed plasma viremia when cART is interrupted. Assessment of HIV-cure strategies using ex vivo culture methods for further understanding of the diversity of reactivated HIV, viral outgrowth, and replication dynamics are enhanced using ultrasensitive digital ELISA based on single-molecule array (Simoa) technology to increase the sensitivity of endpoint detection. In viral outgrowth assays (VOA), exponential HIV-1 outgrowth has been shown to be dependent upon initial virus burst size surpassing a critical growth threshold of 5100 HIV-1 RNA copies. Here, we show an association between ultrasensitive HIV-1 Gag p24 concentrations and HIV-1 RNA copy number that characterize viral dynamics below the exponential replication threshold. Single-genome sequencing (SGS) revealed the presence of multiple identical HIV-1 sequences, indicative of low-level replication occurring below the threshold of exponential outgrowth early during a VOA. However, SGS further revealed diverse related HIV variants detectable by ultrasensitive methods that failed to establish exponential outgrowth. Overall, our data suggest that viral outgrowth occurring below the threshold necessary for establishing exponential growth in culture does not preclude replication competence of reactivated HIV, and ultrasensitive detection of HIV-1 p24 may provide a method to detect previously unquantifiable variants. These data strongly support the use of the Simoa platform in a multi-prong approach to measuring latent viral burden and efficacy of therapeutic interventions aimed at an HIV-1 cure.

## Introduction

While combined antiretroviral therapy (cART) controls Human Immunodeficiency Virus type-1 (HIV-1) replication and largely slows disease progression, life-long adherence to the prescribed cART regimen is required due to the presence of long-lived viral reservoirs capable of resurgence when therapy is interrupted^1–5^. This reservoir is established very early in infection, widely dispersed throughout lymphoid tissues in the body, and remains a major barrier to HIV-1 eradication^6^. Resting CD4+ T cells are a well-characterized cell type shown to hold a portion of the latent HIV reservoir. A number of clinical trials are currently investigating the effects of latency reversal agents and anti-HIV immune expansion on the clearance of these cells^7,8^.

To determine the efficacy of HIV-1 cure strategies, accurate measurement of latently infected cell frequency is of extreme importance. Conventional PCR-based assays offer increased assay processing speed and sensitivity over ex vivo outgrowth methods but are unable to distinguish between competent reservoirs that could rebound in an individual and those that could not^9–11^. Recently, a number of groups have reported improvement in detection of intact proviral genomes using multiple primers and probes over conserved parts of the viral genome^12,13^. However, it is estimated that 30–40% of virus amplified by this method may still be defective, in part due to sequence polymorphisms limiting the successful amplification of the intact provirus^13–15^. At the other extreme are culture-based methods, such as the quantitative viral outgrowth assay (QVOA), which is considered the gold standard for the detection of replication-competent virus. However, interrogation of the QVOA has revealed it underestimates the true size of the latent reservoir due to a portion of proviruses remaining uninduced after a single round of cell stimulation^11,16^.

In the last 15 years, the field of ultrasensitive protein detection has seen significant progress, including development of a ground-breaking single-molecule array (Simoa) technology. This form of digital bead-based immunoassay platform is capable of providing 1000 times improvement in detection limits compared to traditional ELISA^17–20^. A number of studies have used Simoa to detect HIV-1 p24 at femtomolar concentrations in plasma of people living with HIV (PLWH) as well as in studies examining ex vivo induction of virus expression from cART-suppressed individuals^21–25^. The ultrasensitivity of Simoa provides a clear advantage for measuring low levels of inducible, translationally competent virus. However, the high sensitivity also increases the likelihood of overestimating the true size of the reservoir due to production of viral protein from replication-incompetent virus^11,26,27^. Of interest, digital ELISA is now recommended as one of the primary endpoints for measuring levels of steady-state or inducible, translationally competent virus during HIV-cure-directed clinical trials^14^.

Recently, an elegant theoretical and experimental work by Hataye et al.^28^ attempted to determine the necessary conditions driving establishment or collapse of HIV-1 cellular spread in outgrowth culture. In the study, the group acquired time-series data of HIV-1 released from infected cells in order to quantitatively determine the transition to exponential viral growth, a defining characteristic of replication-competent virus. Interestingly, the group determined that release of replication-competent HIV does not always guarantee viral establishment, even with some de novo infection taking place. However, following extensive testing for synergy for viral establishment, the researchers found their outgrowth system supported HIV-1 spread following latency reversal in cases of the initial virus burst surpassing 5100 HIV-1 RNA copies, a critical threshold necessary for the establishment of exponential growth in outgrowth culture. It was determined that above this threshold, on average, there was a sufficient number of released HIV-1 to support synergistic growth, while below that threshold, the virus would typically go extinct. Given that finding, we hypothesized that Simoa technology may be capable of detecting HIV-1 below the critical growth threshold of 5,100 HIV-1 RNA copies. To test this hypothesis, we coupled the QVOA with digital p24 readout and HIV-1 RNA quantification to examine growth kinetics of ultra-low levels of virus in culture supernatants. This approach allowed improved characterization of the diversity of reactivated virus detected by Simoa and provided early detection of replication-competent variants.

## Results

### Defining a robust HIV-1 p24 limit of detection

**Figure 1:**
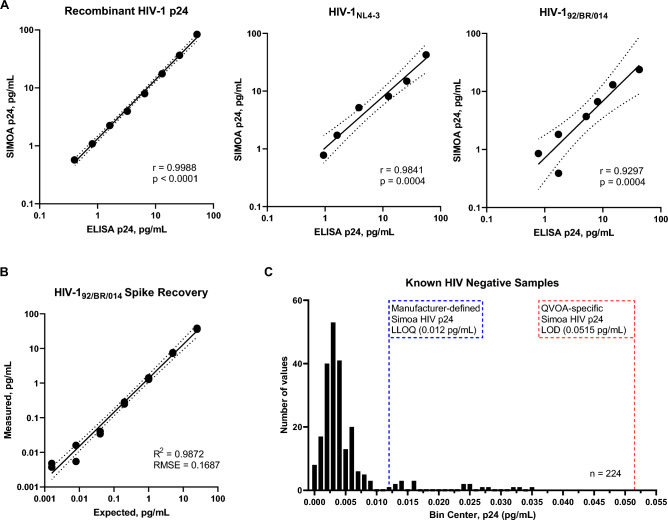
Digital ELISA provides a robust method of detecting HIV-1 p24. (**A**) Correlation of HIV-1 p24 as measured by Simoa and conventional ELISA (Pearson’s correlation coefficient, r). Two replicates were assessed for each dilution set, both for Simoa and conventional ELISA. (**B**) Evaluation of recovery of spiked clinical isolate HIV-1_92/BR/014_ in HIV negatives samples. HIV-1_92/BR/014_ was serially diluted and the assay matrix effect was evaluated by comparing measured concentration by Simoa to the expected (RMSE = root mean square error). Two replicates were assessed for each dilution paramater. (**C**) Frequency Distribution of HIV negative samples. Limit of Detection (LOD) for QVOA-specific digital p24 assay was determined to be 0.0515 pg/mL. To calculate LOD, a Grubb’s outlier test was performed to remove 2 significant outliers from a total of 226 HIV negative samples (Participant 6513-R). The QVOA-specific LOD was calculated by adding three standard deviations (0.005577 pg/mL) of the mean to the maximum p24 value (0.03480 pg/mL) obtained from known HIV negative samples.

In order to foster confidence in ultrasensitive and specific detection of HIV-1 p24 in supernatants of QVOA using the Quanterix Simoa platform, we assessed and adjusted detection limits to address the matrix effects, an important issue during the evaluation of ligand binding assays^29^. To evaluate digital ELISA as a novel QVOA endpoint, we first compared quantification of recombinant HIV-1 p24 protein, HIV-1_NL4-3_, and HIV-1_92/BR/014_ serially diluted in complete media using digital and conventional ELISA approaches. Working within p24 concentration ranges common to both methods, ultrasensitive digital and conventional ELISA provided antigen concentrations that significantly correlated with each other (Fig. 1A), with recombinant protein providing the highest correlation coefficient (r = 0.9988, P < 0.0001). Importantly, HIV-1 culture isolates HIV-1_NL4-3_ and HIV-1_92/BR/014_ also provided strong correlations between quantification methods, suggesting digital ELISA effectively detects and quantifies p24 as part of infectious virions. However, given the greatest observed variability appeared at the low end of the testing range, it was not possible to determine correlations at concentrations below the lower limit of quantitation (LLOQ) of 3.25 pg/mL of p24 antigen for conventional ELISA, generated from the standard curve^30^.

To begin assessing performance of digital ELISA p24 quantification in the presence of long-term cell culture by-products, we performed spike and recovery testing by serially diluting HIV-1_92/BR/014_ into excess supernatants collected from negative control wells of QVOA, which contained allogeneic feeder cells but no participant peripheral blood mononuclear cells (PBMCs). The expected and measured p24 concentrations exhibited significant correlation with a root mean square error (RMSE) of 0.1678 and an R^2^ of 0.9872 (Fig. 1B). Importantly, the values observed were within the acceptable range of 0.008 to 39.5 pg/mL as suggested from the certificate of analysis for the Simoa p24 kit used in the initial assessment analysis (Simoa HIV p24 kit, Lot 500621). This finding provided a strong evidence of digital ELISA sensitivity in the context of culture supernatant matrix.

**Figure 2:**
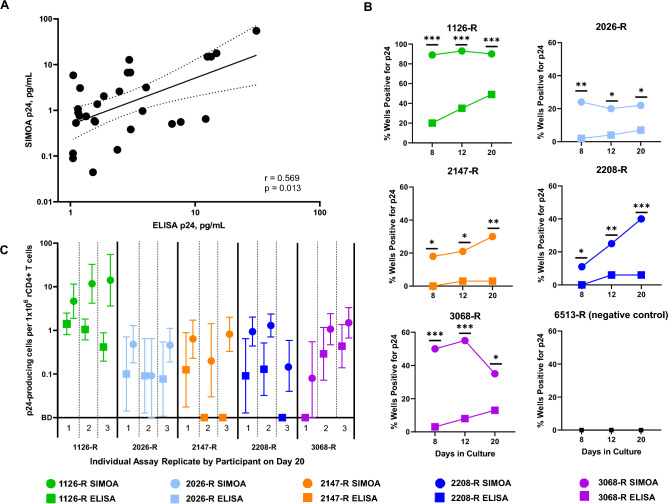
Digital ELISA yields higher reservoir measurements from HIV-1 infected donors and detects HIV-1 p24 at earlier assay time points. (**A**) Correlation of HIV-1 p24 measurements by Simoa and conventional ELISA (Pearson’s correlation coefficient, r) in a set of triplicate QVOAs performed on 5 HIV-1 infected donors. (**B**) Digital ELISA produces significantly more positive wells over the course of the assay duration on different days as found through chi-square analysis, * denotes p < 0.05, ** denotes p < 0.01, and *** denotes p < 0.001. (**C**) Coupling Simoa with QVOA allowed all assays to yield a reservoir measurement while conventional ELISA did not produce a quantifiable measurement in four assays. Reservoir frequencies and associated 95% confidence intervals (error bars) are plotted for each replicate from day 20 of the QVOA culture. Regions, where confidence intervals do not overlap, are significant. *BD* below detection.

To determine a robust and QVOA-specific limit of detection (LOD), using ultrasensitive Simoa HIV p24 measurement, we analyzed a total of 226 supernatants produced during QVOA performed on PBMC from a known HIV-1 seronegative donor (participant 6513-R). A Grubb’s outlier test was performed, and two significant outliers were found and removed from the data set. Although the p24 measured in the majority (92.4%) of the supernatants were below the manufacturer specified LLOQ of 0.012 pg/mL (Fig. 1C) (Simoa HIV p24 Kit, Lot 500802), 17 samples from known HIV-negative QVOA wells yielded p24 antigen concentrations above the manufacturer’s LLOQ, resulting in a false-positive rate of 7.6%. To eradicate false-positive readings and appropriately capture functional sensitivity of digital ELISA, we determined a QVOA-specific LOD of 0.0515 pg/mL by adding three standard deviations of the average p24 from this sample set to the highest concentration of p24 found while testing supernatants from known HIV-negative culture wells. Of interest, a similar value has been reported in a recent study examining the use of digital ELISA in the context of QVOA^31^. The authors determined the LLOQ of the assay and that concentrations at which there was less than 20% total was to be approximately 50 fg/mL, or 0.050 pg/mL. However, given that in our study we did not perform analysis of the total error of the assay^32,33^, we refer to this parameter as the LOD. To confirm use of this new LOD and assess the correlation between digital and conventional ELISA p24 values, we identified and obtained culture supernatants from assays performed for 5 independent HIV-positive participants (1126-R, 2026-R, 2147-R, 2208-R, and 3068-R) assessed by triplicate QVOA as previously reported^34^. Similar to our in vitro findings, we observed a significant correlation between p24 concentrations generated by digital and conventional ELISA when assessing HIV reactivation from ex vivo samples being tested by QVOA (Fig. 2A). In addition, assessment of the negative controls for these assays also served to validate our newly established LOD for the Simoa platform (Fig. 2B).

### Ultrasensitive digital ELISA enhances detection and quantification of HIV reservoirs

One of our primary goals in evaluating ultrasensitive p24 detection was to determine if Simoa would enhance identification of reactivated replication-competent HIV provirus. We measured the concentration of HIV-1 p24 using conventional and digital ELISAs on QVOA supernatants collected from the assays on days 8, 12, and 20^34^. Due to limited culture volumes from previous studies^34^, these analyses were performed on available and previously cryopreserved QVOA supernatants (A wells: 1 × 10^6^ rCD4+ T cells/well). As compared to matched conventional ELISA analysis, the use of digital ELISA with a cutoff of 0.0515 pg/mL (51.5 fg/mL) for binary (positive or negative) QVOA well scoring resulted in significantly more p24-positive wells (by Chi-squared analysis) at each assay time point tested (Fig. 2B). Increasing the percentage of wells scored as p24-positive directly converts to higher frequencies of infectious units per million (IUPM) resting CD4+ T cell measurements as calculated by IUPMStats v1.0^35^. Ultrasensitive digital ELISA allows for detection of femtogram per milliliter concentrations of p24, however, since the infectivity of HIV at such ultra-low concentrations has not been confirmed or disproven, we used “p24-producing cells per million” resting CD4+ T cells to define reactivated HIV-reservoir frequencies (Fig. 2C) rather than IUPM. Incorporating a digital ELISA readout allowed all assays to yield a measurable reactivated HIV reservoir frequency, while standard ELISA did not yield quantifiable measurements in four participant assays (Fig. 2C).

### Ultrasensitive HIV p24 detection reveals novel viral growth kinetics below limits of detection for conventional ELISA

**Figure 3:**
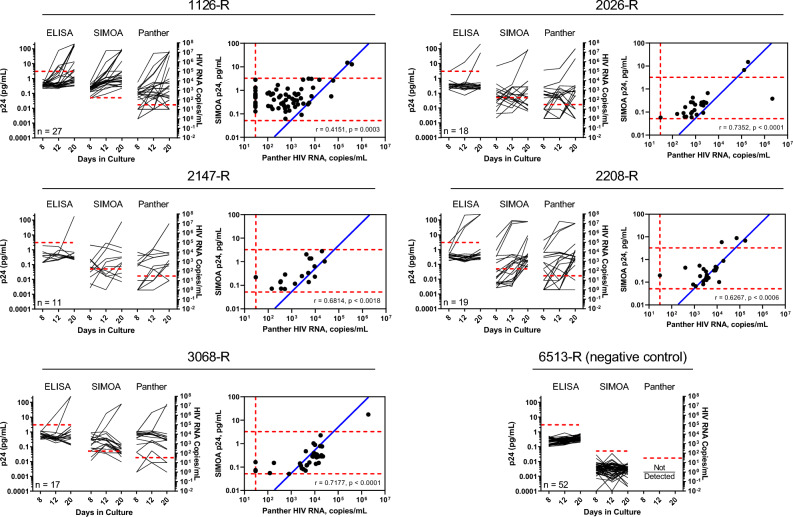
Ultrasensitive HIV-1 p24 and RNA genome detection strongly correlate and reveal growth kinetics at concentrations not detectable by conventional ELISA. Supernatants collected at days 8, 12, and 20 of QVOA that have been published previously^34^ were analyzed for the presence of HIV-1 p24 by standard ELISA, digital ELISA, and HIV-1 RNA by Panther. HIV-1 p24 concentrations measured by digital ELISA and HIV-1 RNA concentrations in QVOA supernatants directly correlate (Pearson’s correlation coefficient, r). Dotted red line indicates Lower Limit of Quantitation for ELISA (3.25 pg/mL p24 antigen) and Panther (30 RNA copies/mL) assays and Limit of Detection for Simoa, (0.0515 pg/mL p24 antigen). Solid blue line indicates the relationship equivalency of 1 pg HIV-1 p24 to 2 × 10^4^ HIV RNA copies, referred to as mature particle correlation (MPC).

To investigate the integrity of virus being detected by ultrasensitive methods, we compared the concentration of HIV-1 p24 protein to quantified RNA genomes present in each supernatant that was also previously assessed by conventional ELISA (Fig. 2 and^34^). The concentration of HIV-1 RNA genomes was quantified using the Aptima HIV-1 Quant Dx Assay (Hologic, PRD-03565)^36–38^ on the Panther system (Hologic, San Diego, CA). As shown in Fig. 3, digital ELISA and Panther analyses performed on QVOA supernatants from assay days 8, 12, and 20 revealed mirrored HIV-1 growth kinetics above and below the limit of detection by conventional ELISA. Cases of HIV-1 p24 and RNA genome concentration increasing, stabilizing, and decreasing were observed across the 92 QVOA cultures that were analyzed. By performing correlation analysis of interpolated data coupled with QVOA-specific LOD, in all five study participants, the concentration of HIV-1 p24 and RNA genomes exhibited a significant positive correlation. We observed a coefficient of correlation range from r = 0.4151 (p = 0.0003) for the participant 1126-R to r = 0.7352 (p < 0.0001) for the participant 2026-R.

It has been previously shown that in the presence of protease inhibitors, which prevent the cleavage of Gag and Gag-Pol protein precursors, virus particles released from HIV-1 infected cells are not infectious and immature^39,40^. A significantly higher amount of HIV p24 Gag protein forms the immature virus core^41^. By tracking the relationship equivalency of 1 pg HIV p24 to 2 × 10^4^ HIV RNA copies, which corresponds to a fully assembled virus particle according to established stoichiometry models^41^, depicted as a solid blue line in Fig. 3 and further referred to as mature particle correlation (MPC), it became possible to identify the defective or immature virus particles compared to the fully mature ones. While all the samples analyzed exhibited deviation from the MPC, we have also observed samples in agreement with MPC in the region close to the Simoa QVOA-specific LOD. Of interest, in participant 1126-R, a large number of samples analyzed contained a higher amount of HIV p24 compared to the RNA genomes, indicating an induction of defective proviruses with retained ability to produce p24 following ex vivo stimulation in the context of viral outgrowth assay. Unsurprisingly, the coefficient of correlation for 1126-R was the lowest among the five study participants analyzed.

Matched, proportional growth kinetics between HIV-1 p24 and RNA genome concentrations over the course of a QVOA provided an adequate dataset to empirically test if the frequency of p24-producing cells per million resting CD4+ T cells changes over time between assay days 8, 12, and 20. Similar to earlier findings^31^, we found that digital ELISA performed on 12-day culture samples provided the greatest average fold-increase in the frequency of p24-producing cells per million (7.01 ± 0.31) as compared to conventional ELISA at day 20 of culture (Table 1S) and that maintaining the cultures beyond 12 days did not significantly increase the number of positive wells identified by either ELISA method for all five study participants from the HIV Reservoir Assay Validation and Evaluation Network (RAVEN). (Chi-square analyses: digital ELISA p > 0.0722, conventional ELISA p > 0.0893). Taken together, these results suggest that digital ELISA effectively shortens the duration of QVOA culturing, resulting in reduction of efforts and costs while measuring latent HIV-1 reservoirs, which is of major importance to the HIV-1 cure agenda^42,43^.

**Figure 4:**
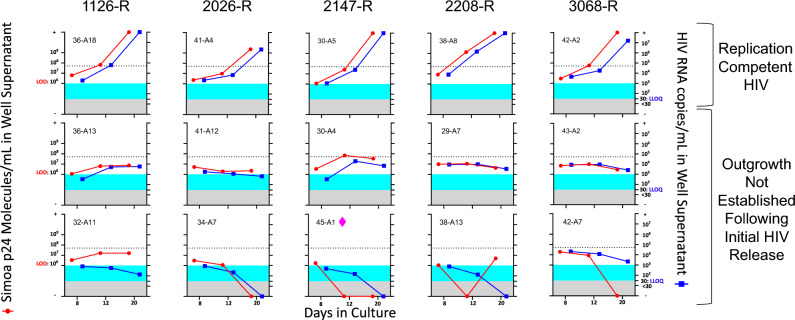
Ultrasensitive methods for detecting HIV-1 p24 and viral RNA provide evidence of HIV-1 reactivation that does not lead to exponential outgrowth. Each panel represents a unique well from an individual QVOA displaying viral kinetics through measurements of either HIV-1 p24 molecules (red-filled circles and red solid lines) or HIV RNA copies (blue-filled squares and blue solid lines) taken at three different time points during the assay. HIV-1 p24 molecules per milliliter axis set to 1000-times the RNA genome axis. The dotted line represents 5,100 HIV RNA copies threshold (converted to 51,000 HIV RNA copies/mL). The light-blue-filled area represents area below the LOD of the digital ELISA (1 × 10^6^ p24 molecules/mL). The grey-filled area represents the area below the lower limit of quantitation (LLOQ) of the Panther Hologic HIV-1 RNA system of 30 HIV RNA copies/mL. For sample 45-A1 (marked with a pink diamond), single-genome sequencing was performed to assess viral replication (Fig. 5).

Given that HIV-1 RNA quantification by Aptima HIV-1 Quant Dx Assay (Fig. 3) showed a clear presence of viral genomes and recent work revealed a viral burst of 5100 HIV RNA copies is needed to establish exponential outgrowth^28^, we examined HIV-1 p24 and RNA genome concentrations and kinetics as it related to viral burst size and subsequent outgrowth. In our QVOA cultures containing reactivated HIV-1 from cART-suppressed study participants, we determined if exponential outgrowth or lack thereof could be explained by the Allee effect, a biological phenomenon characterizing the population establishment as a by-product of synergy among individuals^28,44,45^. Previous studies have demonstrated the number of p24 units needed to form the HIV capsid to range between 1000 and 3000^41,46^. In this study, we assumed 2000 p24 molecules per virion^22,47,48^. With one virion containing two copies of RNA, we set the HIV-1 p24 molecules per milliliter axis to 1000-times the RNA genome axis. Furthermore, following the stoichiometry model^41^ that suggests 1 pg of p24 Gag protein is equivalent to 10^4^ virus particles, we calculated Simoa LOD of 0.0515 pg/m being equal to 1 × 10^6^ p24 molecules/mL (light-blue-filled area in Fig. 4). Lastly, following the conversion of the threshold of 5,100 total HIV RNA copies (value associated with 0.1 mL of culture supernatant)^28^ to 51,000 HIV RNA copies/mL, we discovered this threshold did in fact dictate subsequent viral outgrowth in select samples analyzed (Fig. 4). This observation was particularly evident in cases when digital readout detected HIV p24 concentration approximately equal to or above the threshold (Fig. 4: 36-A18 Day 12, 30-A5 Day 12, and 42-A2 Day 12). Of interest, we also identified a large number of culture wells containing virus incapable of establishing exponential infection, rather, smoldering around the predicted critical value or declining altogether. Taken together, these results suggest that viral supernatants characterized by low-level p24 as detected by ultrasensitive digital ELISA may maintain replication-competent virions but are incapable of establishing exponential replication due to insufficient replication levels following latent HIV-1 provirus reactivation.

### Digital ELISA identifies diverse viral sequences potentially undergoing low-level replication

**Figure 5:**
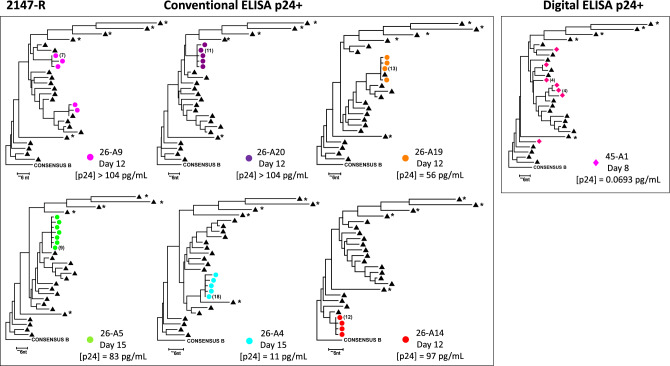
Low concentrations p24 requiring ultrasensitive detection approaches reveal a more diverse repertoire of reactivated HIV-1. Each circle or diamond represents a single, unique RNA genome found through P6-PR-RT single-genome sequencing^80^ performed on QVOA culture supernatants from RAVEN participant 2147-R. Black triangles represent proviral sequences found in mixed PBMC from RAVEN participant 2147-R. Asterisk denotes stop codons. Sequences were processed to visualize 1–2 nucleotide changes.

To characterize the diversity of induced virus found in digital ELISA p24+/conventional ELISA p24- culture supernatants, we performed single-genome sequencing (SGS) on the viral RNA from well 45-A1 of QVOA 45 (2147-R) that failed to establish exponential replication after being detected as p24+ through digital readout (Figs. 4 and 5), as well as on viral RNA from wells of QVOA 26 (2147-R) that successfully established outgrowth. Of interest, six out of seven nodes, identified by SGS analysis of P6-PR-RT on six supernatants exhibiting conventional replication competence, were found to contain between 7 and 18 identical sequences, possibly suggesting induction of replication-competent provirus in expanded T cell clones in vivo^49,50^. Furthermore, select sequences within wells showed the presence of single nucleotide differences from a rake of identical sequences, which is likely indicative of accumulation of new mutations on the background of the identical sequences as a result of RT error following viral replication in the QVOA well, rather than the induction of different proviral clones. In contrast, the single supernatant from well 45-A1 (2147-R) that required digital ELISA for p24 detection (0.0693 pg/mL HIV-1 p24) revealed six nodes consisting of seven unique sequences, indicating reactivation of a larger pool of proviral DNA. In two of these six nodes we observed four identical sequences, potentially suggesting reactivation of replication-competent provirus that failed to establish outgrowth, although a match between a near-full length sequence from QVOA wells and cell-associated HIV RNA or provirus DNA would make for a stronger case. The presence of one node showing single nucleotide differences further suggests a low-level of viral replication within this well. Despite attempts at performing SGS analysis on supernatants from QVOA days 12 and 20 where p24 concentrations ranged from 0.0719 to 2.797 pg/mL, no detectable single-genome sequences were found beyond day 8 of culturing, potentially indicating RNA shearing in long term culture.

### Ultrasensitive p24 digital ELISA coupled with QVOA produces an HIV-1 reservoir measurement in a 15-year follow up sample from a participant of an early HIV CAR T cell clinical trial

Given the commitment of efforts, funding, and samples required to run QVOA for clinical trial evaluation, determining HIV-1 reservoir measurements below assay-specific limits of detection with conventional ELISA can be inconclusive. Initial scoring for HIV-1 reactivation in a reservoir measurement for a > 15 year follow-up sample collected from a study participant of an RV130/514 clinical trial (NCT01013415) through conventional ELISA did not yield a reservoir measurement, however, digital ELISA identified HIV-1 p24 above the LOD in 7 out of 16 supernatants (Fig. 1S). All the measurements collected by digital ELISA exhibited HIV-1 p24 molecule concentrations below the critical threshold of genome copy equivalents required to establish exponential replication, suggesting either inadequate levels of virus reactivation or insufficient levels of replication inside the wells, consequently leading to failure to launch ex vivo exponential outgrowth in culture. Ultimately, the QVOA coupled with digital ELISA produced a reservoir measurement (0.778 p24-producing cells per million resting CD4 T cells with a 95% confidence interval from 0.365 to 1.665) whereas conventional ELISA and integrated HIV-1 DNA assay did not return a quantifiable measurement (Fig. 1S).

## Discussion

With the advent of Simoa technology^17,19,20^, digital p24 ELISA became a novel endpoint for assays measuring HIV reservoir^21,23,31^. However, given high sensitivity of digital ELISA, questions of non-specific background signals, and abundant defective provirus, concerns have arisen around replication competence of low-level virus detected by Simoa. Here, we have described the kinetics and composition of virus particles that fell below the threshold required for ex vivo exponential outgrowth and were detectable only via ultrasensitive HIV-1 detection methods. These characterizations, performed on longitudinal culture supernatants, were accomplished by quantifying HIV-1 p24 molecules using Simoa and RNA genomes using the Panther Aptima platform. As it stands, our study is the first to incorporate the use of Simoa technology in combination with QVOA, high-throughput HIV-1 RNA quantification, and single-genome sequencing to examine the nature of low-level p24 detected by ultrasensitive methods.

Finding strong correlations between virus components (Fig. 3) and a ratio of 2000 HIV-1 p24 molecules to 2 HIV-1 RNA genome copies, as represented by left and right axes in Fig. 4 scaled to 1000 to 1, respectively, may suggest production of appropriately assembled virus particles, rather than unassembled capsid being released from dying cells. As can be expected, across 92 QVOA cultures generated from samples donated by five PLWH, we observed a wide range of replication kinetics including exponential outgrowth, cases where ratios of p24 molecules to RNA genomes suggest assembled viral particles, and instances where ratios of p24 molecules to RNA genome exemplify defective or unassembled virus particles. This latter observation is in line with previous findings showing the significant proportion of genomes having hypermutations and internal deletions^11,51–55^ that could still produce p24. Of interest, a recent study^56^ revealed a large variability among the induced proviruses relying on molecular assays, whereas in our study, we present evidence of the inter- and intra-participant variability of inducible translationally competent provirus.

Similar to other groups^24,31^ who have successfully applied digital p24 readout to estimate the size of the HIV-1 latent reservoir, we observed a number of instances where HIV-1 negative samples from QVOA appeared to be positive upon initial analysis. To solve this issue and to determine a QVOA-specific LLOQ, a modified protocol incorporating Triton X-100 and dilution buffer consisting of casein in PBS and FBS was suggested^24^. Of interest, we report here a comparable LOD without implementing protocol modifications, but rather determined through an in-depth analysis of 226 HIV-1 negative samples that employed previously published methods with minor modifications. This finding suggests that Quanterix manufacturer’s conditions are sufficient for HIV-1 p24 detection and quantification in a viral outgrowth assay. It appears that culture assay matrix is the primary determinant of the LOD variability and warrants further investigation if higher sensitivity is desired.

Importantly, our results directly corroborate recent studies by Hataye et al.^28^ showing that transition to exponential growth, a crucial characteristic of virus fit for rebound, is dependent upon virus burst size exceeding a critical threshold of 5100 HIV RNA copies. In our study, we provide supporting evidence for a critical threshold requirement during ex vivo HIV-1 reactivation, culturing, and outgrowth from cART-suppressed PLWH. Importantly, we detected instances where virus particle concentrations were originally below the critical threshold but subsequently established productive infection, indicating that p24 detected by digital ELISA should not be dismissed. It is important to note that our study did not use viral inhibitors, meaning we were unable to detect the initial burst size without ongoing propagation. Our data demonstrate digital ELISA detects viral antigen below the critical threshold necessary to establish exponential growth, which may indicate ex vivo culture systems are less permissive for HIV-1 reactivation, replication, and spread as compared to lymphatic tissue in PLWH. However, failure to establish ex vivo growth may not reflect the capacity of the released virion to establish replication in the lymphoid tissue where the conditions and spatiotemporal dynamics of HIV propagation may be more favorable^57^. Direct evidence remains to be generated to determine whether digital ELISA is able to detect initial viral bursts in the presence of viral inhibitors and whether virus particles that fail to establish exponential replication ex vivo are intact and competent to rebound in vivo.

Likewise, Panther Aptima HIV-1 Quant Dx Assay uses target-capture transcription-mediated amplification (TMA) and real-time detection mediated through targeting highly conserved regions of HIV-1 polymerase (pol) and long terminal repeat (LTR)^36^, which may suggest that samples with low level p24 concentrations do not represent empty virus particles. Our findings imply a proportion of virus particles measurable only by digital ELISA may be replication-competent, similar to findings using ultrasensitive cell-free HIV-RNA quantification methods^58^. Furthermore, as determined through SGS analysis, outgrowth in Simoa positive wells did not match previously detected variants in addition to revealing more sequence nodes, suggestive of reactivation of a more diverse pool of HIV-1 provirus. Given the background of a diverse proviral population, the two cases where four identical HIV-1 genome sequences were identified likely indicates the induction of replication-competent provirus. Moreover, the presence of single nucleotide differences from these identical sequences could explain smoldering replication below the threshold required for exponential outgrowth.

As it stands, the QVOA, while being labor and resource intensive, provides minimal measurements of the latent reservoir and exponential outgrowth in the QVOA is currently considered the gold standard method to measure the truly replication-competent reservoir. Given the development of less expensive, more sensitive, and higher-throughput assays for the measurement of HIV-1 latent reservoir in clinical trials is of significant importance, there has been remarkable progress in developing improvements to QVOA. Some approaches use alternative stimulation methods^59^, sequencing-based methods^60^, and humanized mice to detect replication-competent virus^61,62^. More recently, based on the findings that effector memory T cells contain a higher proportion of inducible HIV-1^63^, our lab has independently developed and qualified a modified QVOA, termed differentiation QVOA or dQVOA^50^. The assay incorporates a cytokine cocktail aimed at ex vivo differentiation of resting CD4+ T cells into effector memory population prior to global stimulation and viral outgrowth, which increases reactivated and replication-competent reservoir measurements by up to 18-fold while continuing binary well scoring through conventional ELISA. Given digital immunoassay produced an average sevenfold increase in HIV reservoir measurements over conventional ELISA coupled with standard QVOA and a recent discovery of a large proportion of genetically intact HIV-1 proviruses in effector memory CD4+ T cells^55^, coupling digital ELISA with dQVOA may result in further increases in HIV-1 reservoir measurements.

Most of the assays currently used to evaluate HIV-1 persistence rely on HIV-1 DNA or RNA. While such assays are effective in the evaluation of HIV-1 persistence and support in-depth analysis of sequence diversity, copy number, integration diversity, genome intactness, and diversity of RNA species^12,64–67^, they are limited in evaluation of translationally competent reservoirs. Up until recently, the use of assays capable of measuring translation-competent viruses has been limited due to limit of detection not being close to that of nucleic acid-based assays. To the best of our knowledge, Simoa platform is the only ELISA-based system that allows the measuring of HIV Gag protein with the sensitivity comparable to that of nucleic acid-based assays^22,23,68,69^. While replication-competence was not confirmed through these studies, and a definition of HIV reactivation in terms of the quantity of cells capable of producing p24 is not yet established as an endpoint in assessing HIV-1 eradication approaches, it is known that persistent p24 expression contributes to chronic immune activation and HIV-1 pathogenesis^26,70–72^. Simoa provides the ability to monitor p24-producing cells via measuring concentration of HIV-1 p24 in the supernatant at the cell culture level, intracellular level^24^, as well as in plasma and serum^21,22^ when the conventional immunoassay would not produce a quantifiable result. Further, in vitro replication competence has been argued to be an ineffective substitute for in vivo rebound capability^11,16^. Of interest, an ultrasensitive immunoassay for Simian Immunodeficiency Virus (SIV) p27 capsid has recently been reported^73^, which, in its present form, can be used to identify SIV p27 Gag in culture supernatants using ex vivo cells from SIV- or Simian HIV (SHIV)-infected non-human primates on suppressive cART combined with the ability to assess phenotypes of post-treatment controllers. Moreover, a recent 27-fold improvement in sensitivity^74^ of the digital HIV-1 p24 assay and increased throughput and miniaturization^75^ of the assay are poised to position Simoa platform as a unique tool to measure viral production at sub-picogram levels.

We recognize that our study has limitations. Because we only conducted Panther HIV-1 RNA analysis on select samples, we cannot provide an estimate of the frequency of improperly packaged virions, which is likely a result of defective provirus reactivation. However, this issue needs to be balanced against budgetary considerations and additional technical challenges. Increased sensitivity, at the expense of increases in costs and time, could result from incorporating supernatant and proviral sequencing. This approach would allow one to establish a proportion of defective and intact proviruses capable of reaching the critical growth threshold and successfully establishing infection. In our analyses, single-genome sequencing of culture supernatants suggests viral replication early on in the assay. However, given the lack of exponential outgrowth in subsequent days, it is possible the culture conditions were not optimal for the induction of a sufficient number of replication competent proviruses to establish transition to exponential outgrowth. Alternatively, it could be that in QVOA cultures positive by conventional ELISA the HIV variant with the best fitness ends up outcompeting the rest of the induced variants, whereas, in a QVOA culture positive by Simoa, the HIV variants, while more diverse, are of lower fitness. Subsequently, such variants are not able to cross the outgrowth threshold and fail to establish productive spread in culture. Additionally, we cannot rule out recombination of different viral variants in the same well giving rise to a spreading infection. A recent study which used autologous antibodies to control for the confounding effects of recombination was able to further discern composition of the latent reservoir^76^. Future studies coupling this approach with the use of Simoa platform may lead to further advances in understanding HIV latency.

In conclusion, our data show that ultrasensitive HIV-1 p24 ELISA can be a valuable tool in a multi-prong approach at characterizing and quantifying levels of translationally competent and possibly replication-competent virus when conventional methods do not produce a quantifiable measurement. Additional modifications such as immunoprecipitation^69^ and decreasing capture beads combined with high efficiency bead analysis^74^ could yield further increases in assay sensitivity bringing it to the levels of nucleic acid testing with the advantage of measuring release of mature HIV virions. As such, the use of a digital ELISA readout capable of detecting and quantifying HIV-1 p24 below the threshold necessary for the establishment of outgrowth in culture could be a key component in a multi-faceted approach to measure effectiveness of interventions for treatment and prevention in HIV-cure-directed clinical trials.

## Methods

### Study participants

Cryopreserved PBMCs from six HIV-1 infected participants on suppressive cART and one HIV-1 negative donor were obtained for the purpose of this study. One HIV-1 infected donor was from the RV130/514 trial (NCT01013415). Five HIV-1 infected and one HIV-1 negative donors were from the HIV Reservoir Assay Validation and Evaluation Network (RAVEN). This sample set included a wide range in magnitude of replication-competent reservoirs in order to evaluate inter-lab repeatability and intra-lab precision analysis^34^. Clinical characteristics and HIV-1 reservoir sizes for the five RAVEN participants have been published previously^9^. Infection status was confirmed through appropriate Antigen/Antibody and Nucleic Acid Testing.

### Ethics statement

Sampling of the RAVEN cohort was overseen and approved by the UCSF Committee on Human Research (IRB #10-03244). Work related to RV130/514 trial (NCT01013415) was approved by the Walter Reed Army Medical Center, Walter Reed National Military Medical Center, Walter Reed Army Institute of Research and the Uniformed Services University institutional review boards. All research was conducted in accordance with relevant guidelines and regulations. All study participants provided written, informed consent.

### QVOA

The Quantitative Viral Outgrowth Assay (QVOA) was performed as previously described^30^ with minor modifications. In brief, cryopreserved PBMCs from HIV-1 infected or HIV-1 negative donors were thawed and cultured overnight in complete media (R10) consisting of RPMI 1640 Medium with GlutaMAX™ supplement (Gibco, 61870-036) supplemented with 10% heat-inactivated fetal bovine serum (Peak Serum, PS-FB1), 1% penicillin and 1% streptomycin (Gibco, 15140-122). For the RAVEN cohort^34,77^, CD4+ T cells were isolated from bulk PBMC by negative selection (Human CD4+ Isolation Kit, Miltenyi Biotec, 130-096-533) followed by resting CD4+ (rCD4+) T lymphocytes enrichment through depletion of cells expressing HLA-DR (anti-HLA-DR MircoBeads, Miltenyi Biotec, 130-046-101), CD25 (CD25 Microbead Kit, Miltenyi Biotec, 130-092-983), and CD69 (CD69 Microbead Kit, Miltenyi Biotec, 130-092-355). For the participant from the RV130/514 trial, resting CD4+ T cells were enriched through negative, magnetic bead separation with the use of EasySep™D Magnetic Particles (Stem Cell, 17962 and 19250). For all assays, the purity of enrichment was assessed through flow cytometry analysis. For each assay, rCD4+ T cells were seeded in up to 22 replicates of 1 × 10^6^ cells per well (A wells), 2 replicates of 2 × 10^5^ cells per well (B wells), 2 replicates of 4 × 10^4^ cells per well (C wells), 2 replicates of 8 × 10^3^ cells per well (D wells), 2 replicates of 1.6 × 10^3^ cells per well (E wells), 2 replicates of 320 cells per well (F well), and 2 replicates of negative control wells without rCD4+ T cells (G wells) in R10 supplemented with 100 IU/mL recombinant human IL-2 (R&D Systems, 202-IL), 1–1.2% T cell growth factor (TCGF) (generated as described previously^30^), and 0.5 µg/mL PHA (ThermoFisher Scientific, R30852801 or Sigma-Aldrich, L1668-5MG). Allogeneic mixtures of γ-irradiated PBMCs from HIV-seronegative donors (Bioreclamation or New York Blood Center) provided co-stimulation in a 10:1 ratio in each well. Following overnight incubation for a minimum of 16 h, PHA was washed off through a media change and CD8-depleted (ThermoFisher Scientific, 11147D), PHA-activated lymphoblasts (target cells) were added for viral amplification. The assays were maintained for up to 20 days according to previously published protocol^30^ with the additional steps of supernatant collection on media and cell replacement days. Following completion of the assay, p24 antigen was quantified in supernatants from each well by either conventional ELISA (PerkinElmer, NEK050B001KT) per manufacturer’s instructions or ultrasensitive digital ELISA as described below. Conventional ELISA positivity was scored as positive or negative based on a cut-off HIV-1 p24 concentration of ≥ 3.25 pg/mL.

### Ultrasensitive digital immunoassay for HIV-1 p24

HIV-1 p24 in QVOA culture supernatants was quantified using the Simoa HIV p24 Kit (Quanterix, Product Number 102215, Lot Number 500621 for the initial assessment of the platform and Lot Number 500802 for supernatant screen) on the Simoa HD-1 platform (Quanterix Simoa HD-1 1.5.16006.30001 × 64) as per the manufacturer’s instructions. In brief, QVOA culture supernatants, standard curve calibrators, and quality control samples were brought to room temperature, briefly vortexed to ensure homogenization, and centrifuged at 10,000 RCF for 5 min at room temperature to remove insoluble particulates prior to use. A total of 350 µl of each culture supernatant, reference calibrators, complete media, or quality controls were loaded into the 96-well plate (supplied with the Simoa HD-1 Disk Kit, Quanterix 100227-2) and sealed using X-Pierce Sealing Films (Excel Scientific, XP-100). Without customization, the 2-step HIV-1 p24 HD-1 assay definition was utilized to analyze two replicates of each sample. Reference calibrators for the establishment of the standard curve and two quality control samples of low and high concentration were included in each of the runs to ensure consistent performance of the digital assay throughout the study. Moreover, the same lot of Simoa HIV p24 Kit was used for all the samples spanning multiple assays. Post-run quality control checks included verification of the standard curve acceptance criteria (R^2^ > 0.98), confirmation of the standard curve, quality controls, sample CVs (< 20%), ambient temperature and humidity monitoring during the run, adjustment of assay definition concentrations to that of the kit lot, in addition to assessment of any relevant errors during the analysis (e.g. too much fluorescence scored as positive and fluorescence below the standard curve scored as negative) as per manufacturer’s instructions. Four-parameter logistic (4PL) regression fitting, 1/y^2^ weighted, was used to estimate the concentration of p24 in the culture supernatants using the manufacturer’s software analysis. Wells were considered positive for the presence of p24 if the concentration was above 0.0515 pg/mL.

### Digital ELISA performance evaluation and determination of the limit of detection (LOD) for QVOA endpoint

Recombinant HIV-1 p24 protein was obtained from the conventional ELISA kit (Perkin Elmer, NEK050B001KT). The following reagents were obtained through the NIH AIDS Reagent Program, Division of AIDS, NIAID, NIH: Human Immunodeficiency Virus-1 92/BR/014, ARP-1753, contributed by UNAIDS Network for HIV Isolation and Characterization and Human Immunodeficiency Virus 1 (HIV-1), Strain NL4-3 Infectious Molecular Clone (pNL4-3), ARP-2852, contributed by Dr. M. Martin^78^. HIV-1 p24 protein and cultured viruses were serially diluted into complete media R10 and assessed by conventional and digital ELISA. Additionally, HIV-1 92/BR/014 was serially diluted into supernatant collected from QVOA negative control wells (assay matrix) and analyzed by digital ELISA. Finally, a total of 226 HIV-1 negative samples collected from three independently performed QVOAs on one HIV-1 negative participant were analyzed on Quanterix instrument to determine frequency distribution of HIV negative samples. These data were then used to calculate a limit of detection (LOD) of the digital ELISA in the context of QVOA. Grubb’s outlier test was performed to remove two significant outliers. LOD was subsequently calculated by adding three standard deviations of the mean to the maximum p24 value.

### HIV-1 RNA high-throughput quantification

HIV-1 RNA in QVOA culture supernatants was quantified on the fully automated Panther system using the Aptima HIV-1 Quant assay with a lower limit of quantitation of 30 copies/mL and an upper limit of quantitation of 10,000,000 copies/mL (Hologic, San Diego, CA). Due to low volume available for each sample, 0.3 mL of culture supernatant was diluted threefold with Aptima specimen diluent (Hologic, PRD-03003) prior to testing using the 1:3 option on the Panther system, wherein the instrument software automatically reports the neat concentration by applying the dilution factor.

### Single-genome sequencing

Single-genome sequencing (SGS) of HIV-1 p6-PR-RT was performed as previously described^79,80^. Sequences were aligned using ClustalW. Neighbor-joining phylogenetic analyses were performed using MEGA6. Trees were rooted on the consensus sequence of the subtype B.

### Total and integrated HIV DNA measurement

Total HIV DNA levels were measured by quantitative PCR as previously described^81^. Briefly, cell pellets were digested using a Proteinase K lysis buffer. Total HIV-1 DNA was quantified using specific primers situated in the 5’- LTR-gag sequence and integrated DNA was amplified using Alu-gag specific primers. The PCR products were detected by specific probes and the results were normalized to the number of copies of the CD3 gene (2 copies per cell).

### Data analysis

All statistical analysis was performed using GraphPad Prism v9.3.1. Grubbs test was performed to remove significant outliers. Simple linear regression was performed to determine goodness of fit and two-tailed P value was obtained to determine significance for correlation analysis. Frequencies of the infectious units per million (IUPM) were calculated using IUPMStats v1.0 Infection Frequency Calculator^35^.

## Supplementary Information

Supplementary Information.

## Data Availability

All relevant data that support the findings of this study are available from the corresponding authors upon reasonable request. HIV sequences used for phylogenetic analysis have been deposited to GenBank with accession numbers OQ866407-OQ866521 and OQ920078-OQ920090.

## References

[1] Walensky RP (2006). The survival benefits of AIDS treatment in the United States. J. Infect. Dis..

[2] Chun TW (1998). Early establishment of a pool of latently infected, resting CD4(+) T cells during primary HIV-1 infection. Proc. Natl. Acad. Sci. USA.

[3] Whitney JB (2014). Rapid seeding of the viral reservoir prior to SIV viraemia in rhesus monkeys. Nature.

[4] Colby DJ (2018). Rapid HIV RNA rebound after antiretroviral treatment interruption in persons durably suppressed in Fiebig I acute HIV infection. Nat. Med..

[5] Finzi D (1997). Identification of a reservoir for HIV-1 in patients on highly active antiretroviral therapy. Science.

[6] Chun TW, Moir S, Fauci AS (2015). HIV reservoirs as obstacles and opportunities for an HIV cure. Nat. Immunol..

[7] Henderson LJ, Reoma LB, Kovacs JA, Nath A (2020). Advances toward curing HIV-1 infection in tissue reservoirs. J. Virol..

[8] Barr L, Jefferys R (2019). A landscape analysis of HIV cure-related clinical trials and observational studies in 2018. J. Virus Erad..

[9] Eriksson S (2013). Comparative analysis of measures of viral reservoirs in HIV-1 eradication studies. PLoS Pathog..

[10] Bruner KM (2016). Defective proviruses rapidly accumulate during acute HIV-1 infection. Nat. Med..

[11] Ho YC (2013). Replication-competent noninduced proviruses in the latent reservoir increase barrier to HIV-1 cure. Cell.

[12] Bruner KM (2019). A quantitative approach for measuring the reservoir of latent HIV-1 proviruses. Nature.

[13] Gaebler C (2019). Combination of quadruplex qPCR and next-generation sequencing for qualitative and quantitative analysis of the HIV-1 latent reservoir. J. Exp. Med..

[14] Abdel-Mohsen M (2020). Recommendations for measuring HIV reservoir size in cure-directed clinical trials. Nat. Med..

[15] Kinloch NN (2021). HIV-1 diversity considerations in the application of the Intact Proviral DNA Assay (IPDA). Nat. Commun..

[16] Hosmane NN (2017). Proliferation of latently infected CD4(+) T cells carrying replication-competent HIV-1: Potential role in latent reservoir dynamics. J. Exp. Med..

[17] Rissin DM, Walt DR (2006). Digital concentration readout of single enzyme molecules using femtoliter arrays and Poisson statistics. Nano Lett..

[18] Rissin DM, Walt DR (2006). Digital readout of target binding with attomole detection limits via enzyme amplification in femtoliter arrays. J. Am. Chem. Soc..

[19] Wilson DH (2016). The Simoa HD-1 analyzer: A novel fully automated digital immunoassay analyzer with single-molecule sensitivity and multiplexing. J. Lab. Autom..

[20] Rissin DM (2010). Single-molecule enzyme-linked immunosorbent assay detects serum proteins at subfemtomolar concentrations. Nat. Biotechnol..

[21] Passaes CPB (2017). Ultrasensitive HIV-1 p24 assay detects single infected cells and differences in reservoir induction by latency reversal agents. J. Virol..

[22] Chang L (2013). Simple diffusion-constrained immunoassay for p24 protein with the sensitivity of nucleic acid amplification for detecting acute HIV infection. J. Virol. Methods.

[23] Passaes C (2021). Ultrasensitive detection of p24 in plasma samples from people with primary and chronic HIV-1 infection. J. Virol..

[24] Wu G (2017). HDAC inhibition induces HIV-1 protein and enables immune-based clearance following latency reversal. JCI Insight.

[25] Wu G (2021). Gag p24 is a marker of human immunodeficiency virus expression in tissues and correlates with immune response. J. Infect. Dis..

[26] Imamichi H (2020). Defective HIV-1 proviruses produce viral proteins. Proc. Natl. Acad. Sci. USA.

[27] Fisher K (2022). Plasma-derived HIV-1 virions contain considerable levels of defective genomes. J. Virol..

[28] Hataye JM (2019). Principles governing establishment versus collapse of HIV-1 cellular spread. Cell Host Microbe.

[29] US Food and Drug Administration. Bioanalytical method validation guidance for industry. *US Department of Health and Human Services, Food and Drug Administration, Center for Drug Evaluation and Research, and Center for Veterinary Medicine* (2018).

[30] Laird GM, Rosenbloom DI, Lai J, Siliciano RF, Siliciano JD (2016). Measuring the frequency of latent HIV-1 in resting CD4(+) T cells using a limiting dilution coculture assay. Methods Mol. Biol..

[31] Stuelke EL (2020). Measuring the inducible, replication-competent HIV reservoir using an ultra-sensitive p24 readout, the digital ELISA viral outgrowth assay. Front. Immunol..

[32] Pierson-Perry JF, Vaks JE, Durham AP (2012). Evaluation of Detection Capability for Clinical Laboratory Measurement Procedures; Approved Guideline.

[33] Armbruster DA, Pry T (2008). Limit of blank, limit of detection and limit of quantitation. Clin. Biochem. Rev..

[34] Rosenbloom DIS (2019). Assessing intra-lab precision and inter-lab repeatability of outgrowth assays of HIV-1 latent reservoir size. PLoS Comput. Biol..

[35] Rosenbloom DI (2015). Designing and interpreting limiting dilution assays: General principles and applications to the latent reservoir for human immunodeficiency virus-1. Open Forum Infect. Dis..

[36] *Hologic. Aptima HIV-1 Quant Dx Assay.*, https://www.hologic.com/package-inserts/diagnostic-products/aptima-hiv-1-quant-dx-assay-us-ivd.

[37] Nair SV (2016). Aptima HIV-1 Quant Dx–A fully automated assay for both diagnosis and quantification of HIV-1. J. Clin. Virol..

[38] Stone M (2018). Comparison of detection limits of fourth- and fifth-generation combination HIV antigen-antibody, p24 antigen, and viral load assays on diverse HIV isolates. J. Clin. Microbiol..

[39] Wang SW, Aldovini A (2002). RNA incorporation is critical for retroviral particle integrity after cell membrane assembly of Gag complexes. J. Virol..

[40] Kaplan AH (1993). Partial inhibition of the human immunodeficiency virus type 1 protease results in aberrant virus assembly and the formation of noninfectious particles. J. Virol..

[41] Briggs JA (2004). The stoichiometry of Gag protein in HIV-1. Nat. Struct. Mol. Biol..

[42] Ndung'u T, McCune JM, Deeks SG (2019). Why and where an HIV cure is needed and how it might be achieved. Nature.

[43] International, A. S. S. W. G. o. H. I. V. C. (2012). Towards an HIV cure: A global scientific strategy. Nat. Rev. Immunol..

[44] Dennis B (2002). Allee effects in stochastic populations. Oikos.

[45] Drake JM, Lodge DM (2006). Allee effects, propagule pressure and the probability of establishment: Risk analysis for biological invasions. Biol. Invasions.

[46] Summers MF (1992). Nucleocapsid zinc fingers detected in retroviruses: EXAFS studies of intact viruses and the solution-state structure of the nucleocapsid protein from HIV-1. Protein Sci..

[47] Piatak M (1993). High levels of HIV-1 in plasma during all stages of infection determined by competitive PCR. Science.

[48] Marozsan AJ (2004). Relationships between infectious titer, capsid protein levels, and reverse transcriptase activities of diverse human immunodeficiency virus type 1 isolates. J. Virol..

[49] Bui JK (2017). Proviruses with identical sequences comprise a large fraction of the replication-competent HIV reservoir. PLoS Pathog..

[50] Wonderlich ER (2019). Effector memory differentiation increases detection of replication-competent HIV-l in resting CD4+ T cells from virally suppressed individuals. PLoS Pathog..

[51] Hiener B (2017). Identification of genetically intact HIV-1 proviruses in specific CD4(+) T cells from effectively treated participants. Cell Rep..

[52] Cohn LB (2018). Clonal CD4(+) T cells in the HIV-1 latent reservoir display a distinct gene profile upon reactivation. Nat. Med..

[53] Wagner R (2000). Rev-independent expression of synthetic gag-pol genes of human immunodeficiency virus type 1 and simian immunodeficiency virus: Implications for the safety of lentiviral vectors. Hum. Gene Ther..

[54] Blissenbach M, Grewe B, Hoffmann B, Brandt S, Uberla K (2010). Nuclear RNA export and packaging functions of HIV-1 Rev revisited. J. Virol..

[55] Duette G (2022). The HIV-1 proviral landscape reveals that Nef contributes to HIV-1 persistence in effector memory CD4+ T cells. J. Clin. Investig..

[56] Simonetti FR (2020). Intact proviral DNA assay analysis of large cohorts of people with HIV provides a benchmark for the frequency and composition of persistent proviral DNA. Proc. Natl. Acad. Sci. USA.

[57] Strain MC, Richman DD, Wong JK, Levine H (2002). Spatiotemporal dynamics of HIV propagation. J. Theor. Biol..

[58] Richman DD (2019). Replication competence of virions induced from CD4+ lymphocytes latently infected with HIV. Retrovirology.

[59] Kuzmichev YV (2017). A CD3/CD28 microbead-based HIV-1 viral outgrowth assay. J. Virus Erad..

[60] Lee SK (2017). Quantification of the latent HIV-1 reservoir using ultra deep sequencing and primer ID in a viral outgrowth assay. J. Acquir. Immune Defic. Syndr..

[61] Charlins P (2017). A humanized mouse-based HIV-1 viral outgrowth assay with higher sensitivity than in vitro qVOA in detecting latently infected cells from individuals on ART with undetectable viral loads. Virology.

[62] Metcalf Pate KA (2015). A murine viral outgrowth assay to detect residual HIV type 1 in patients with undetectable viral loads. J. Infect. Dis..

[63] Kulpa DA (2019). Differentiation into an effector memory phenotype potentiates HIV-1 latency reversal in CD4(+) T cells. J. Virol..

[64] Procopio FA (2015). A novel assay to measure the magnitude of the inducible viral reservoir in HIV-infected individuals. EBioMedicine.

[65] Butler SL, Hansen MS, Bushman FD (2001). A quantitative assay for HIV DNA integration in vivo. Nat. Med..

[66] O'Doherty U, Swiggard WJ, Jeyakumar D, McGain D, Malim MH (2002). A sensitive, quantitative assay for human immunodeficiency virus type 1 integration. J. Virol..

[67] Yukl SA (2018). HIV latency in isolated patient CD4(+) T cells may be due to blocks in HIV transcriptional elongation, completion, and splicing. Sci. Transl. Med..

[68] Cabrera C, Chang L, Stone M, Busch M, Wilson DH (2015). Rapid, fully automated digital immunoassay for p24 protein with the sensitivity of nucleic acid amplification for detecting acute HIV infection. Clin. Chem..

[69] Wu G (2021). Improved detection of HIV Gag p24 protein using a combined immunoprecipitation and digital ELISA method. Front. Microbiol..

[70] Imamichi H (2016). Defective HIV-1 proviruses produce novel protein-coding RNA species in HIV-infected patients on combination antiretroviral therapy. Proc. Natl. Acad. Sci. USA.

[71] Pollack RA (2017). Defective HIV-1 proviruses are expressed and can be recognized by cytotoxic T lymphocytes, which shape the proviral landscape. Cell Host Microbe.

[72] Stevenson EM (2021). HIV-specific T cell responses reflect substantive in vivo interactions with antigen despite long-term therapy. JCI Insight.

[73] Swanstrom AE (2018). Ultrasensitive immunoassay for simian immunodeficiency virus p27(CA). AIDS Res. Hum. Retroviruses.

[74] Kan CW (2020). Digital enzyme-linked immunosorbent assays with sub-attomolar detection limits based on low numbers of capture beads combined with high efficiency bead analysis. Lab. Chip.

[75] Levinger C (2021). An ultrasensitive planar array p24 Gag ELISA to detect HIV-1 in diverse biological matrixes. Sci. Rep..

[76] Bertagnolli LN (2020). Autologous IgG antibodies block outgrowth of a substantial but variable fraction of viruses in the latent reservoir for HIV-1. Proc. Natl. Acad. Sci. USA.

[77] Stone M (2020). Assessing suitability of next-generation viral outgrowth assays as proxies for classic QVOA to measure HIV-1 latent reservoir size. J. Infect. Dis..

[78] Adachi A (1986). Production of acquired immunodeficiency syndrome-associated retrovirus in human and nonhuman cells transfected with an infectious molecular clone. J. Virol..

[79] Kearney MF (2016). Origin of rebound plasma HIV includes cells with identical proviruses that are transcriptionally active before stopping of antiretroviral therapy. J. Virol..

[80] Palmer S (2005). Multiple, linked human immunodeficiency virus type 1 drug resistance mutations in treatment-experienced patients are missed by standard genotype analysis. J. Clin. Microbiol..

[81] Vandergeeten C (2014). Cross-clade ultrasensitive PCR-based assays to measure HIV persistence in large-cohort studies. J. Virol..

