# Supplementary material for: Application of ultrasensitive digital ELISA for p24 enables improved evaluation of HIV-1 reservoir diversity and growth kinetics in viral outgrowth assays
Source: Sci Rep. 2023 Jul 6;13:10958. doi: 10.1038/s41598-023-37223-9 (PMC10326067; doi:10.1038/s41598-023-37223-9)
Supplement: Supplementary file 1 — Supplementary Information. [file 41598_2023_37223_MOESM1_ESM.pdf]

Supplementary Materials for

Application of Ultrasensitive Digital ELISA for p24 Enables Improved Evaluation of HIV-1 Reservoir Diversity and Growth Kinetics in Viral Outgrowth Assays

Yury V. Kuzmichev[1,2] *, Carol Lackman-Smith[2], Sonia Bakkour[3,4], Ann Wiegand[5], Michael J. Bale[5, ¤a], Andrew Musick[5], Wendy Bernstein[6,7], Naomi Aronson[6,7], Julie Ake[8], Sodsai Tovanabutra[8], Mars Stone[3,4], Roger G. Ptak[2], Mary F. Kearney[5], Michael P. Busch[3,4], Elizabeth R. Wonderlich[2], Deanna A. Kulpa[1,9] *

[1] Division of Microbiology and Immunology, Emory National Primate Research Center, Emory University, Atlanta, GA, USA
[2] Department of Infectious Disease Research, Southern Research, Frederick, MD, USA.
[3] Vitalant Research Institute, San Francisco, CA, USA.
[4] Department of Laboratory Medicine, University of California San Francisco, San Francisco, CA, USA
[5] HIV Dynamics and Replication Program, NCI at Frederick, NIH, Frederick, MD, USA.
[6] Uniformed Services University, Bethesda, MD, USA.
[7] Walter Reed National Military Medical Center, Bethesda, MD, USA.
[8] U.S. Military HIV Research Program, Walter Reed Army Institute of Research, Silver Spring, MD, USA
[9] Department of Pathology and Laboratory Medicine, Emory University School of Medicine, Atlanta, GA, USA
¤a Current Address: Laboratory of Epigenetics and Immunity, Department of Pathology and Laboratory Medicine, Weill Cornell Medicine, New York, NY, USA

Corresponding authors:

*Deanna A. Kulpa, Email: deanna.kulpa@emory.edu
*Yury V. Kuzmichev, Email: yury.v.kuzmichev@emory.edu

This PDF file includes:

Figure 1S
Table 1S

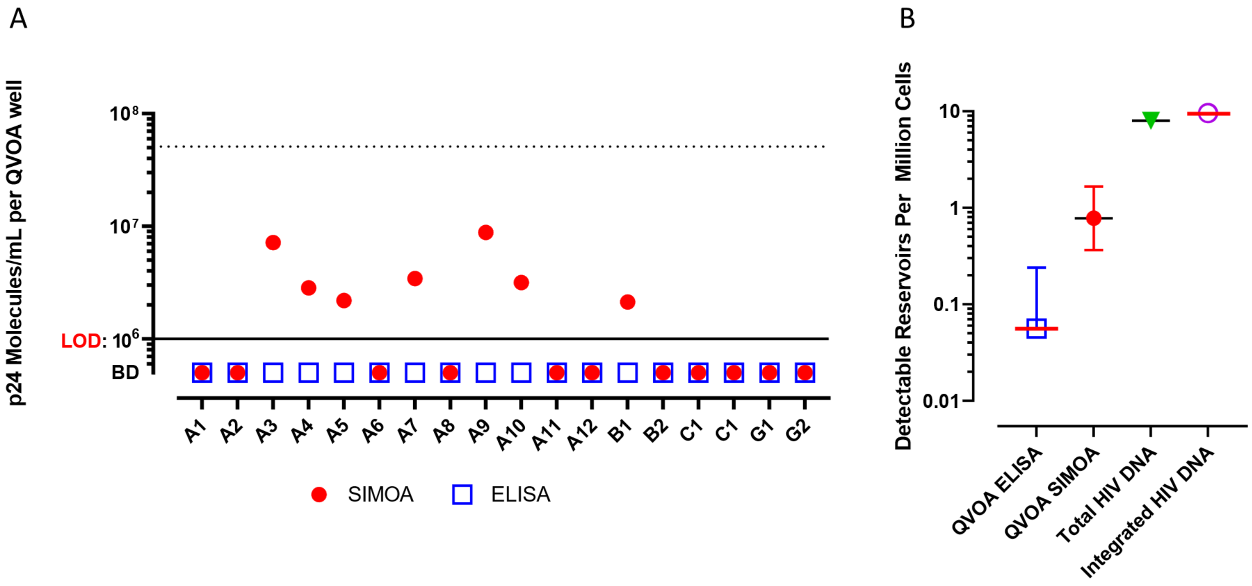

Figure 1S. Ultrasensitive p24 detection provides reservoir measurements where conventional approaches fail. (A). Digital ELISA increases the proportion of QVOA wells found to contain reactivated HIV-1 in samples from RV130/514 clinical trial participant 28. HIV-1 p24 concentrations determined through digital ELISA were converted from pg/mL to molecules/mL. Open blue squares represent p24 concentrations found to be below the limit of detections for conventional ELISA. Red-filled circles reveal the concentration of p24 molecules as determined through digital ELISA. Dotted line represents viral outgrowth threshold (p24 molecule/mL equivalent of 51,000 RNA copies/mL). Solid line represents the limit of detection (LOD) of digital ELISA. (B). Ultrasensitive digital ELISA and total HIV DNA yield measurable reservoir sizes where conventional ELISA and integrated HIV DNA do not produce a quantifiable measurement. Open symbols represent reservoir measurements below the assay-specific limits of detection (LOD, red bar). Total HIV DNA LOD = 7.98 copies/million CD4+ T cells. Integrated HIV DNA LOD = 9.60 copies/million CD4+ T cells.

RAVEN ID	Mean Fold Change - Digital p24/Day 20 ELISA		
	Day 8	Day 12	Day 20
1126-R (n=3)	5.48 (±0.82)	15.57 (±0.79)	10.60 (±0.71)
2026-R (n=3)	4.07 (±0.25)	3.41 (±0.51)	3.81 (±0.65)
2147-R (n=3)	3.38 (±1.17)	3.09 (±0.45)	4.88 (±0.63)
2208-R (n=3)	1.70 (±0.65)	4.44 (±0.83)	8.85 (±0.86)
3068-R (n=3)	15.54 (±1.47)	8.55 (±1.10)	3.40 (±1.12)
**Average**	**6.03 (±0.55)**	**7.01 (±0.31)**	**6.31 (±0.41)**

Table 1S. Digital p24 assay produces 6-7-fold higher frequencies of p24-producing cells per million compared to conventional ELISA. Comparison of the frequency of p24-producing cells per million measured by digital p24 at each time point versus by ELISA on day 20.
